# Parameter optimization for proton density fat fraction quantification in skeletal muscle tissue at 7 T

**DOI:** 10.1007/s10334-024-01195-2

**Published:** 2024-08-06

**Authors:** Katharina Tkotz, Paula Zeiger, Jannis Hanspach, Claudius S. Mathy, Frederik B. Laun, Michael Uder, Armin M. Nagel, Lena V. Gast

**Affiliations:** 1 Institute of Radiology, University Hospital Erlangen, Friedrich-Alexander-Universität Erlangen-Nürnberg (FAU), Erlangen, Germany; 2 Division of Medical Physics in Radiology, German Cancer Research Centre (DKFZ), Heidelberg, Germany

**Keywords:** Fat–water separation (FWS), Proton density fat fraction (PDFF), Dixon MRI, 7 Tesla, Skeletal muscle tissue

## Abstract

**Objective:**

To establish an image acquisition and post-processing workflow for the determination of the proton density fat fraction (PDFF) in calf muscle tissue at 7 T.

**Materials and methods:**

Echo times (TEs) of the applied vendor-provided multi-echo gradient echo sequence were optimized based on simulations of the effective number of signal averages (NSA*). The resulting parameters were validated by measurements in phantom and in healthy calf muscle tissue (*n* = 12). Additionally, methods to reduce phase errors arising at 7 T were evaluated. Finally, PDFF values measured at 7 T in calf muscle tissue of healthy subjects (*n* = 9) and patients with fatty replacement of muscle tissue (*n* = 3) were compared to 3 T results.

**Results:**

Simulations, phantom and in vivo measurements showed the importance of using optimized TEs for the fat–water separation at 7 T. Fat–water swaps could be mitigated using a phase demodulation with an additional B_0_ map, or by shifting the TEs to longer values. Muscular PDFF values measured at 7 T were comparable to measurements at 3 T in both healthy subjects and patients with increased fatty replacement.

**Conclusion:**

PDFF determination in calf muscle tissue is feasible at 7 T using a chemical shift-based approach with optimized acquisition and post-processing parameters.

**Supplementary Information:**

The online version contains supplementary material available at 10.1007/s10334-024-01195-2.

## Introduction

Various pathologies such as neuromuscular disorders [1–3] or sarcopenia [4] are accompanied by progressive fatty replacement of muscle tissue especially in skeletal muscles. Thus, a non-invasive determination of the muscular fat fraction can provide valuable information about the state of disease as well as potential therapeutic effects. Magnetic resonance imaging (MRI) offers a method for measuring the proton density fat fraction (PDFF). The PDFF shows the fraction of MR signal from ^1^H protons bound to lipid molecules relative to the MR signal of the entire tissue, thereby providing a quantitative measure that is proportional to the fat content in the examined tissue. The calculation of the PDFF is typically based on fat–water separation (FWS). This is a commonly used MRI technique based on the chemical shift between water and fat to achieve independent image contrasts for ^1^H signal originating from water and lipid molecules.

The initial approach for FWS proposed by Dixon separates the signal of water and fat based on the signal phase of two measurements performed at echo times (TEs) corresponding to in-phase and opposed-phase conditions [5]. Since then, several approaches have been suggested to further improve the chemical shift-based FWS; for example, by including more echoes [6, 7] or using advanced post-processing schemes incorporating inhomogeneities of the main magnetic field B_0_, the $\text{T}_{2}^{*}$ decay, and multiple fat peaks in the underlying signal models [8, 9]. In general, FWS based on the complex MR signal was shown to be more robust than magnitude-based FWS [10, 11]. Especially, the Iterative Decomposition of Water and Fat with Echo Asymmetry and Least-Squares Estimation (IDEAL) was established for fast, robust and multiresolution separation [9, 12, 13]. Furthermore, a Graph Cut Algorithm was designed for robust FWS in the presence of large B_0_ field inhomogeneities [10, 14].

In the recent years, the use of ultra-high field (UHF) MRI systems (B_0_ ≥ 7 T) has increased, also for imaging skeletal muscle tissue [15]. While the increased signal-to-noise ratio (SNR) enables a higher spatial resolution and better depiction of fine structures in anatomical imaging, the use of UHF systems is particularly beneficial for advanced MRI techniques such as X-nuclei MRI/MRS [16–18] or chemical exchange saturation transfer imaging [19], which usually suffer from low SNR and/or low spectral resolutions. While such metabolic imaging techniques can provide additional insights into (pathological) skeletal muscle tissue, their image contrast is often influenced by fatty replacement of muscle tissue [20–23]. Thus, it is highly desirable to include a stable and reproducible PDFF quantification into the corresponding measurement protocols for skeletal muscle tissue at 7 T. However, optimization of chemical shift-based fat quantification, especially in skeletal muscle tissue, has been mostly performed at more clinically established field strengths such as 1.5 T or 3 T [3, 4, 24–26].

Although basically the same FWS techniques can be applied at UHF, several additional challenges occur when performing PDFF measurements at 7 T. Compared to lower field strengths, the chemical shift between fat and water is increased, which requires more careful optimization of the acquisition parameters to ensure optimal sampling of the fat and water phase oscillations. In particular, shorter TEs and echo time differences are required for optimal sampling efficiency, which can often not be realized due to hardware and sequence restrictions. In addition, inhomogeneities in the main magnetic field B_0_ increase linearly with the field strength. Overall, the B_0_ estimation of the FWS algorithm becomes less robust at higher field strengths. An erroneous B_0_ estimation can result in opposite assignment of the fat and water signal components, a so-called fat–water swap, which leads to inverted PDFF estimation. Therefore, the use of an FWS algorithm that is robust with respect to B_0_ inhomogeneities and/or additional strategies to overcome fat–water swaps are particularly important at 7 T. Finally, increasing vibrational eddy currents may increase phase errors and thereby cause artifacts.

Therefore, the aim of this work was to optimize the acquisition and post-processing parameters for chemical shift-based FWS and PDFF determination in skeletal muscle tissue at 7 T. We used broadly available vendor-provided sequences together with publicly accessible post-processing schemes to provide an approach that can be easily reproduced. The impact of the echo time choice under the given hardware and software constraints on resulting fat quantification was investigated using simulations, phantom experiments and in vivo imaging of the calf. In addition, several methods to correct phase errors arising at 7 T and minimize fat–water swaps were evaluated. Finally, PDFF values in different regions of calf muscle tissue measured at 7 T were compared to corresponding values measured at 3 T.

## Materials and methods

### NSA* simulation

To optimize the TEs used for FWS under the given constraints at 7 T, and assess the influence of the initial echo time (TE_1_) and the echo time difference (ΔTE) on the measured PDFF values, the concept of effective number of signal averages (NSA*) was exploited [6]. In general, the NSA* defines the noise efficiency for the parameter estimation by the FWS. In other words, it describes how accurately the signal parameters (water and fat signal, off-resonances, and potentially the $\text{T}_{2}^{*}$ decay) can be estimated from the phase information between the water and fat signals contained in the acquired echoes. The NSA* for a parameter’s estimate ${\widehat{p}}_{k}$ is given by the ratio of the variance of the parameter ${\sigma }^{2}\left({p}_{k}\right)$ within the measured data and the variance of its estimate ${\sigma }^{2}\left({\widehat{p}}_{k}\right)$ [7]

$$NS{A}^{*}\left({\widehat{p}}_{k}\right)=\frac{{\sigma }^{2}\left({p}_{k}\right)}{{\sigma }^{2}\left({\widehat{p}}_{k}\right)} .$$

For instance, choosing ΔTE such that the phase relation between water and fat signal is the same for all TEs leads to redundant signal information. In this case, a separation of the fat and water signal is impossible and thus, ${\sigma }^{2}\left({\widehat{p}}_{k}\right)$ becomes infinite and NSA* is zero. In contrast, ideally the data from all TEs would provide independent signal information. In this case, the NSA* would be equal to the number of acquired TEs providing a SNR of the estimates that is equal to the signal average over the initially recorded images. The NSA* was first introduced for 3-point Dixon imaging with symmetric echoes [6], and further extended to account also for field inhomogeneities and $\text{T}_{2}^{*}$ decay at asymmetric TEs [27, 28]. For an efficient noise performance, the NSA* should be preferably high. Neglecting $\text{R}_{2}^{*}$, the maximum achievable value corresponds to the number of acquired echoes. However including $\text{R}_{2}^{*}$ in the FWS lowers the achievable maximal NSA*. A low NSA* corresponds to a high variance of the estimates for the fat and water signals and thus, potentially results in an erroneous PDFF estimation. The theoretical NSA* for both 3 T and 7 T FWS was simulated using the ISMRM fat–water toolbox [27, 28] implemented in Matlab (Matlab2019b, The MathWorks). For FWS including estimation of the signal phases and field map, ${\sigma }^{2}\left({p}_{k}\right)$ cannot be calculated directly from the signal equation. Instead the algorithm utilizes a generalized formulation by replacing ${\sigma }^{2}\left({p}_{k}\right)$ with the minimum variance of ${p}_{k}$ under the assumption that it is the only unknown model parameter. The minimum variance of ${p}_{k}$ and ${\sigma }^{2}\left({\widehat{p}}_{k}\right)$ were calculated utilizing the Cramér–Rao lower bounds. For the NSA* simulation, PDFF values were varied from 0 to 100% assuming a single fat peak at –3.3 ppm and an $\text{R}_{2}^{*}$ value of 50 Hz. Six equidistant echoes were used for which TE_1_ and ΔTE were varied between 0.5 ms to 5 ms (step size: 0.05 ms).

### MRI examinations

MRI measurements were conducted at a whole-body 3 T MR system (phantom and healthy volunteers: Magnetom Prisma, Siemens Healthineers, Erlangen, Germany; patients: Magnetom Vida, Siemens Healthineers, Erlangen, Germany) and a whole-body 7 T MR system (Magnetom Terra, Siemens Healthineers, Erlangen, Germany). At 3 T, a 15-channel ^1^H knee coil (Quality Electrodynamics, Mayfield Village, OH, USA) was used for measurements in phantom and healthy volunteers and an 18-channel ^1^H body coil in combination with a 32-channel ^1^H spine coil (both Siemens Healthineers, Erlangen, Germany) for patient measurements. At 7 T, a 28-channel ^1^H knee coil (Quality Electrodynamics, Mayfield Village, OH, USA) was utilized for all measurements. To obtain multi-peak fat models for peanut oil and adipose tissue, single-voxel MR spectroscopy measurements were performed using a STEAM sequence at 7 T (acquisition parameters: TE = 20 ms, TR = 4000 ms, TM = 10 ms, bandwidth (BW) = 4000 Hz, flip angle (FA) = 90°, voxel size = 10 × 10 × 10 mm^3^, 75 averages, acquisition time T_Acq_ = 5 min 20 s). For the FWS image acquisition, a 3D FLASH Volumetric Interpolated Breath-hold Examination (VIBE) GRE sequence with six equidistant echoes acquired in monopolar readout was used (acquisition parameters see Table 1). To reduce T_1_ bias in the PDFF estimation, a small flip angle of 3° was chosen (compare theoretical considerations provided in Supplementary Information Fig. S1). For the comparison of PDFF values with low or high NSA* and the measurements with varying readout direction, a slightly lower in plane resolution with a voxel size of 1.6 × 1.6 × 5 mm^3^ was utilized. For both field strengths, TE_1_ and ΔTE were chosen to achieve a low/high theoretical NSA* based on the simulation results. To mitigate errors arising in the phase of the first echo due to eddy currents at 7 T, a slightly longer TE_1_ at unchanged ΔTE was additionally evaluated. Furthermore, the four available phase encoding directions (R-L, L-R, A-P and P-A) were compared regarding the resulting eddy current artifacts and quantitative PDFF values.

**Table 1:** Acquisition parameters of the applied 3D FLASH VIBE GRE sequence for FWS. Echo times were chosen for both field strengths to achieve a low/high theoretical NSA* as combination of initial echo (TE_1_) and echo time difference (ΔTE)

Parameter	3 T	7 T
TE_1_ [ms]	2.2	1.9
TE_1_ [ms] shifted	-	2.2
$\Delta$TE [ms] high NSA*	3.2	2.3
$\Delta$TE [ms] low NSA*	2.24	2.0
# TE	6	
TR [ms]	21	
BW [Hz/Px]	500	
FA [°]	3	
Slices	16	
Voxel size [mm^3^]	1.0 × 1.0 × 5.0	
FoV read [mm]	200	
FoV phase [%]	100	
T_Acq_ [min:s]	1:11	

### Data post-processing

FWS based on the complex MR signal of the six acquired echoes was performed using the Graph Cut algorithm [10] available within the ISMRM fat–water toolbox implemented in Matlab. 50 iterations considering ${R}_{2}^{*}$ (101 values from 0 to 400 Hz) and off-resonances (401 values from – 300 to 300 Hz) were conducted. A 9-peak and 8-peak fat model were used for the evaluation of the phantom and in vivo data, respectively (see Table 2). The corresponding peak frequencies and amplitudes were extracted from the acquired MR spectra using jMRUI [29, 30]. A hard phase correction was applied, and manually selected peaks were fitted using the Advanced method for Accurate Robust and Efficient Spectral fitting (AMARES) [31].

**Table 2:** Frequency shift (relative to water) and relative amplitudes of the fat spectrum in the peanut oil-agarose phantom and in the subcutaneous fat of the calf acquired at 7 T (*n* = 12)

		P1	P2	P3	P4	P5	P6	P7	P8	P9
phantom	freq.[ppm]	− 3.93	− 3.51	− 3.22	− 2.79	− 2.57	− 2.05	− 0.73	− 0.51	0.52
	rel. ampl	0.082	0.623	0.015	0.096	0.048	0.009	0.026	0.018	0.083
in vivo	freq.[ppm]	− 3.66	− 3.24	− 2.91	− 2.49	− 2.27	− 1.71	− 0.39		0.78
	rel. ampl	0.083	0.688	0.010	0.098	0.042	0.006	0.013		0.060

To further mitigate fat–water swaps, separate B_0_ field maps were acquired using the same GRE sequence, but optimized (in phase) TEs for B_0_ mapping (TE_1_ = 2.04 ms, TE_2_ = 4.08 ms, TR = 8.1 ms). Based on the resulting off-resonance frequencies Δ*f*, a demodulation of the measured complex signal $S\left({TE}_{n}\right)$ of each of the six echoes was performed via multiplication with a phase term [32, 33]:

$$\widehat{S}\left({TE}_{n}\right)=S\left({TE}_{n}\right)\, {e}^{-i 2\pi \Delta f{ TE}_{n}}=\rho \, {e}^{i 2\pi \left(\varphi -\Delta f\right) {TE}_{n}},$$

with $\rho$ describing the measured signal amplitude and $\varphi$ the measured signal phase. The demodulated signal $\widehat{S}\left({TE}_{n}\right)$ was then transferred to the Graph Cut algorithm. 

To investigate the influence of phase errors on the PDFF calculations, the results obtained using the Graph Cut algorithm were compared to the results of using a mixed fitting approach. Starting from the results of the Graph Cut algorithm, this approach adds an additional voxel-wise fitting that regards only the magnitude information of the first echo, but the full complex information of all further echoes [34].

Based on the separated magnitude fat and water images, $\left|F\right|$ and $\left|W\right|,$ resulting from the Graph Cut algorithm, the PDFF was calculated for each voxel according to:

$$PDFF=\frac{\left|F\right|}{\left|W\right|+\left|F\right|}$$

For quantitative analysis, mean and standard deviation of the PDFF were calculated in defined regions of interest (ROIs).

### Phantom and in vivo measurements

**Fig. 1:**
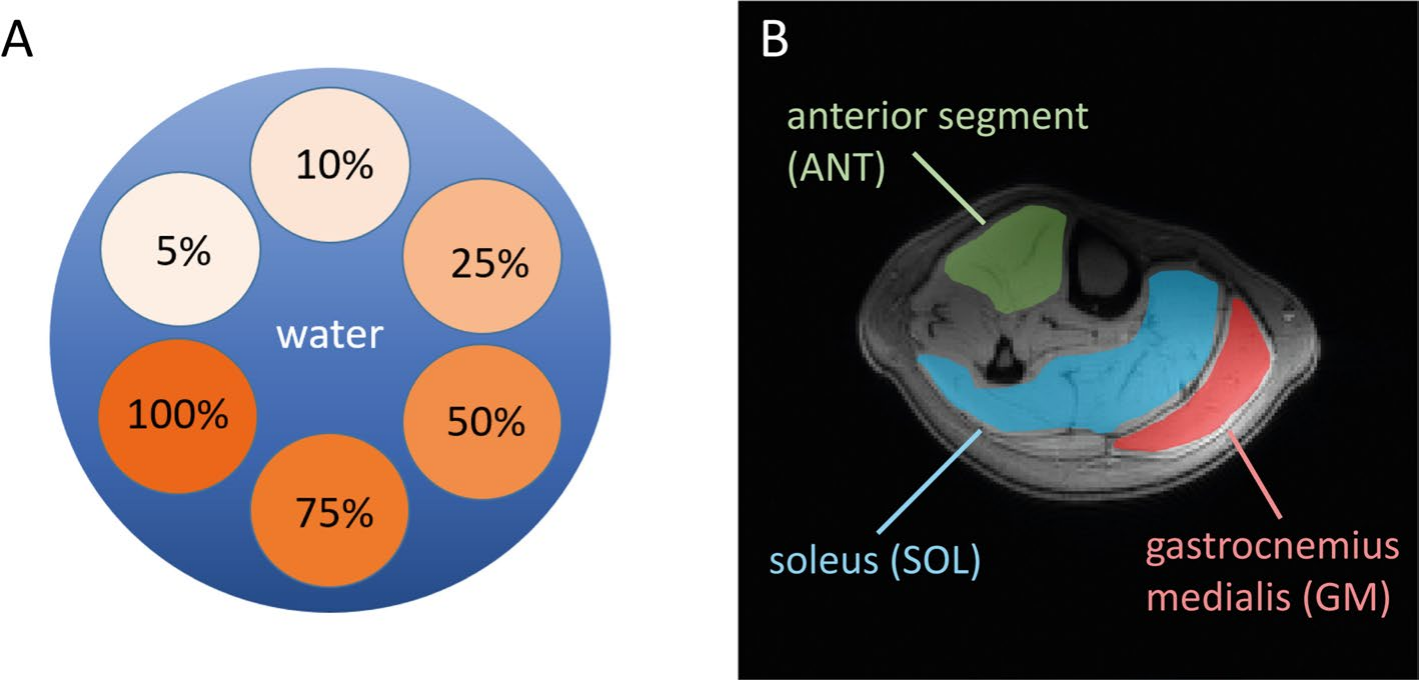
Schematic view in transversal plane of the fat fraction phantom: six vials containing different volume fat fractions (VFFs) of peanut oil and agarose gel were created, covering a broad range of fat fractions (**A**). Regions of interest (ROIs) in the transversal plane of the calf (**B**). Three muscle groups (ANT, SOL, GM) were chosen to represent the muscle tissue. The mean value and standard deviation of the proton density fat fraction (PDFF) was calculated within these ROIs for quantitative evaluation

To assess the accuracy of the PDFF determination, measurements were performed using a phantom containing six vials with emulsions of peanut oil and agarose (3% m/v) [35]. The corresponding volume fat fractions (VFFs) were 5%, 10%, 25%, 50%, 75% and 100% (see Fig. 1). The vials were mounted in a plastic holder and placed within a water-filled bottle. In the phantom, ROIs were placed within each of the six vials, and the resulting experimentally obtained fat fractions were compared to the expected values. The agreement between VFF and measured PDFF was specified by the coefficient of determination (*R*^2^), which gives the variability of the linear fit model, and the mean absolute error (MAE) defined as

$$MAE=\frac{1}{6}\sum_{k=1}^{6}\left|{VFF}_{k}-{PDFF}_{k}\right|$$

In addition, a water-only phantom was used to examine phase errors at 7 T.

For evaluation of the PDFF acquisition and post-processing approach in vivo, the calves of 12 healthy volunteers (6 male, 6 female, mean age 24 ± 2 years) were scanned at 3 T and 7 T during the same day. The measurements were performed with approval of the local ethics committee. Written informed consent was obtained from all subjects. Using the optimized acquisition protocol, the PDFF was additionally measured in nine healthy subjects (5 male, 4 female, mean age 32 ± 19 years) and three patients with hypokalemic periodic paralysis (CACNA1S, 2 female, 1 male, mean age 59 ± 3) at 3 T and 7 T. PDFF within the calf was evaluated in three muscle groups: an anterior segment (ANT), consisting of the tibialis anterior and the extensor digitorum longus, the soleus (SOL) and the gastrocnemius medialis (GM) muscles (see Fig. 1). The ROIs were drawn on the center slice of the 3 T and 7 T measurement individually. To ensure accurate co-localization at both scanners, the center of the coil and of the image stack were positioned at the largest circumference of the calf.

### Statistical analysis

Differences between PDFF values of individual muscle regions as well as between different acquisition/post-processing approaches were statistically assessed using a one-way ANOVA test and a multi-comparison with Bonferroni correction performed in Matlab. The correlation between PDFF values measured at 3 T and 7 T was investigated using the spearman rank coefficient. A *p* value below 0.05 was considered significant.

## Results

**Fig. 2:**
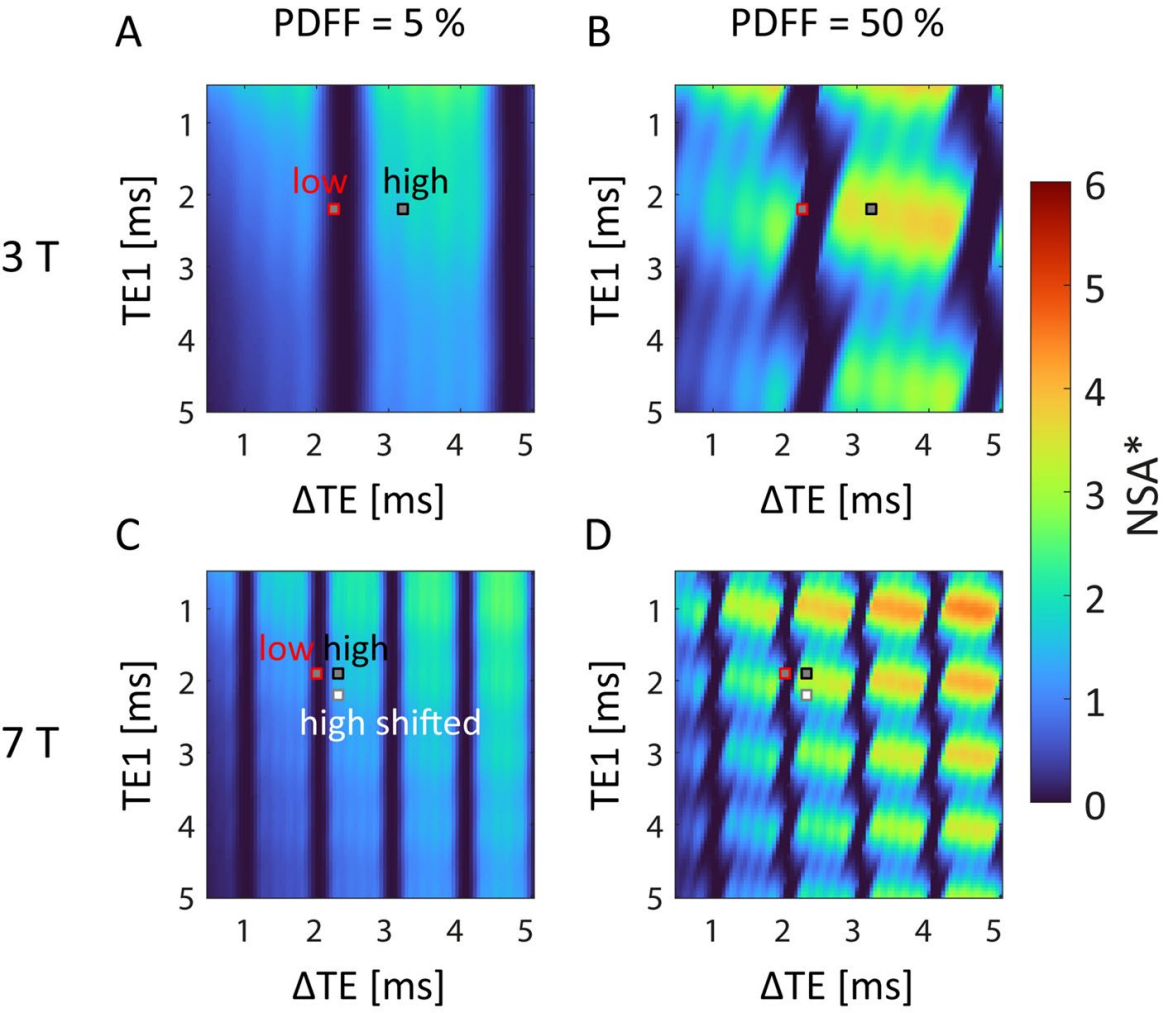
Simulated NSA* for the estimated water magnitude images resulting from FWS depending on initial echo time (TE1) and echo time difference (ΔTE) at B_0_ = 3 T (**A**, **B**) and B_0_ = 7 T (**C**, **D**). Simulations were performed assuming a single-peak fat model considering only the dominant fat peak at −3.3 ppm as well as a relaxation rate of $\text{R}_{2}^{*}$ = 50 Hz for PDFF values of 5% (**A**, **C**) and 50% (**B**, **D**). For the higher PDFF, the dependence of the simulated NSA* on TE1 and the maximum achievable NSA* values were higher. With increasing field strength, the areas with high NSA* became smaller and closer together. Echo combinations for low NSA* (red boxes) and high NSA* (black boxes) which could be realized using the vendorprovided GRE sequence at 3 T and 7 T are marked. At 7 T, an additional TE combination with slightly longer TE1 (marked with white box) was also used to mitigate phase errors. Corresponding plots for PDFF values of 0%, 25%, 50%, 75% and 100% for all parameter estimates at B_0_ = 3 T and 7 T are shown in Supplementary Information Figs. S2 and S3, respectively

Figure 2 illustrates the NSA* simulation results for the water-only magnitude maps after FWS for two different PDFFs (5%, 50%) at 3 T and 7 T. Simulation results for the other parameters of the signal model (water phase, fat magnitude and phase, off-resonance and ${R}_{2}^{*}$) can be found in the Supplementary Information (Figs. S2 and S3). Furthermore, simulation results for additional PDFFs (0%, 25%, 50%, 100%) as well as different ${R}_{2}^{*}$ values (0 Hz, 50 Hz, 100 Hz, 150 Hz) are shown in Supplementary Information Figures S4 and S5. For increasing PDFF the maximum achievable NSA* for estimation of water-only maps became higher than for lower PDFFs. The dependence of the NSA* on TE_1_ was maximal at medium PDFFs. At 7 T, the parameter ranges (TE_1_ and $\Delta$TE) leading to high NSA* values decreased compared to 3 T, and an overall stronger dependence on the chosen TEs was observed. A higher $\text{R}_{2}^{*}$ led to a reduced NSA* for longer TE_1_ as well as ΔTE. The ΔTE corresponding to the Nyquist frequency for sampling of the fat–water phase oscillation was about 1.2 ms at 3 T and 0.5 ms at 7 T. A ΔTE below this value with high NSA* would be optimal to minimize the risk of fat–water swaps.

**Fig. 3:**
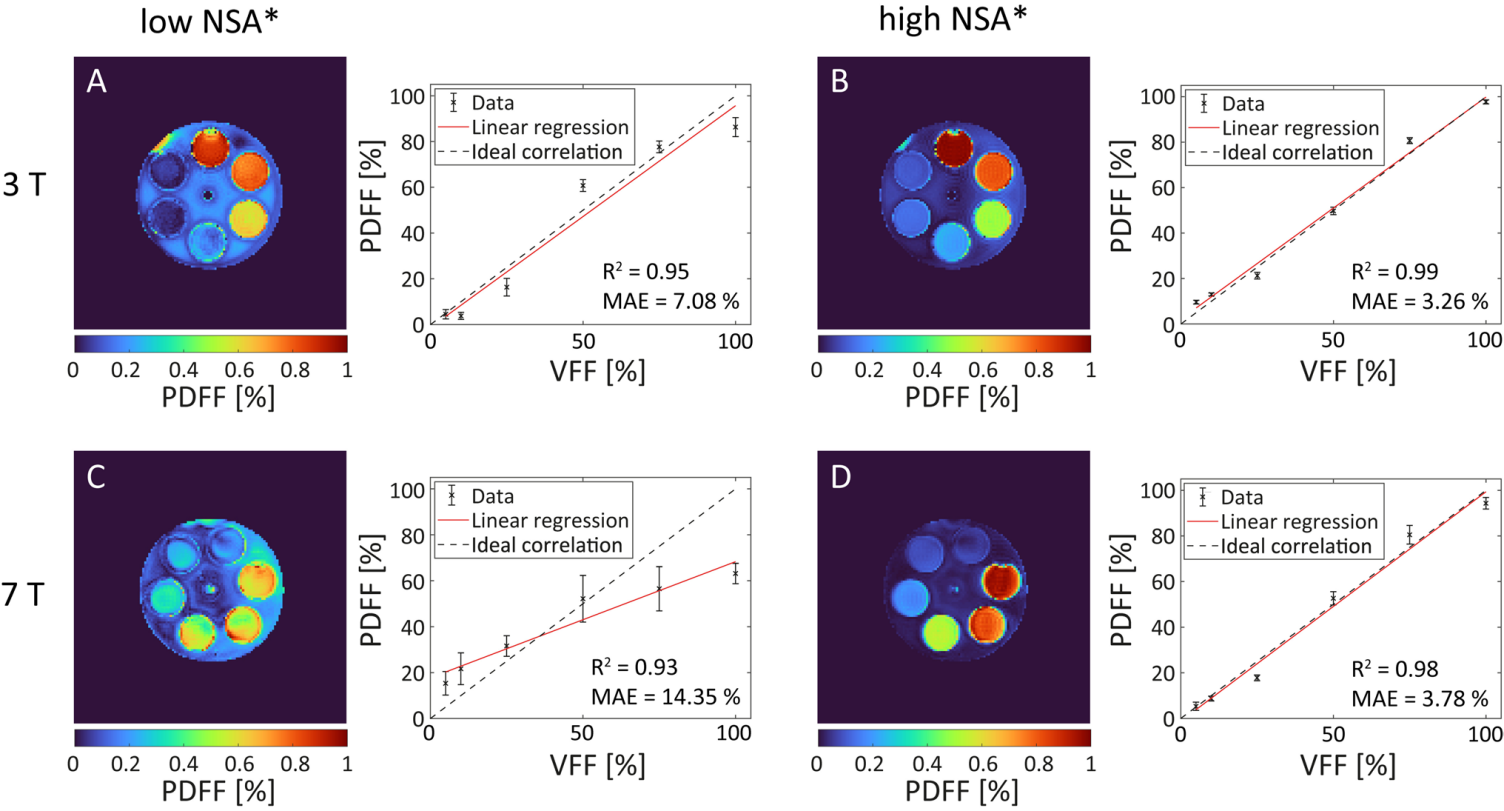
Experimental verification of the NSA* simulation at B_0_ = 3 T (**A**, **B**) and B_0_ = 7 T (**C**, **D**) with the agarose-peanut oil phantom. PDFF maps calculated based on complex data acquired with low (**A**, **C**) and high theoretical NSA* (**B**, **D**) in phase encoding direction R-L. For all measurements, plots of the PDFF mean values within the six vials against the corresponding VFFs are shown. The coefficient of determination (R^2^) rates the linearity of the data in terms of the shown regression line, and the mean absolute error (MAE) measures the agreement between PDFF and VFF. At both field strengths, the MAE was lower for high NSA* echo times than for low NSA* echo times. Especially at 7 T, the low NSA* led to highly deviating PDFF values from the corresponding VFF. In addition, the coefficient of determination was smaller for low NSA* and the linear regression line at 7 T strongly deviated from the ideal correlation. Using high NSA* echo times, the coefficient of determination of the data was very high for both field strengths and the regression line and the ideal correlation matched well

For the experimental verification, feasible TE combinations considering the constraints of the used vendor-provided GRE sequence leading to either low or high theoretical NSA* were chosen based on the simulations, as marked in Fig. 2. With the used vendor-provided sequence, it was not possible to choose $\Delta$TE short enough to achieve optimal sampling of the fat–water phase oscillation for the desired image resolution and bandwidth, neither at 3 T nor at 7 T. While at 3 T the used $\Delta$TE with high NSA* was more than twice the $\Delta$TE for optimal sampling, it was more than four times the optimal $\Delta$TE at 7 T. To evaluate the quantitative results, PDFF maps of the peanut-oil phantom acquired at 3 T and 7 T were evaluated (Fig. 3). PDFF maps acquired using TE combinations leading to low theoretical NSA* strongly overestimated the PDFF within the surrounding water both at 3 T and 7 T. In addition, the agreement between measured PDFF and VFF of the vials was low for low NSA* measurements, as reflected by a high mean deviation between these two values (MAE_3T_ = 7.1%, MAE_7T_ = 14.4%). For the high NSA* TE combinations, the PDFF maps showed a high agreement with the expected VFFs (MAE_3T_ = 3.3%, MAE_7T_ = 3.8%).

**Fig. 4:**
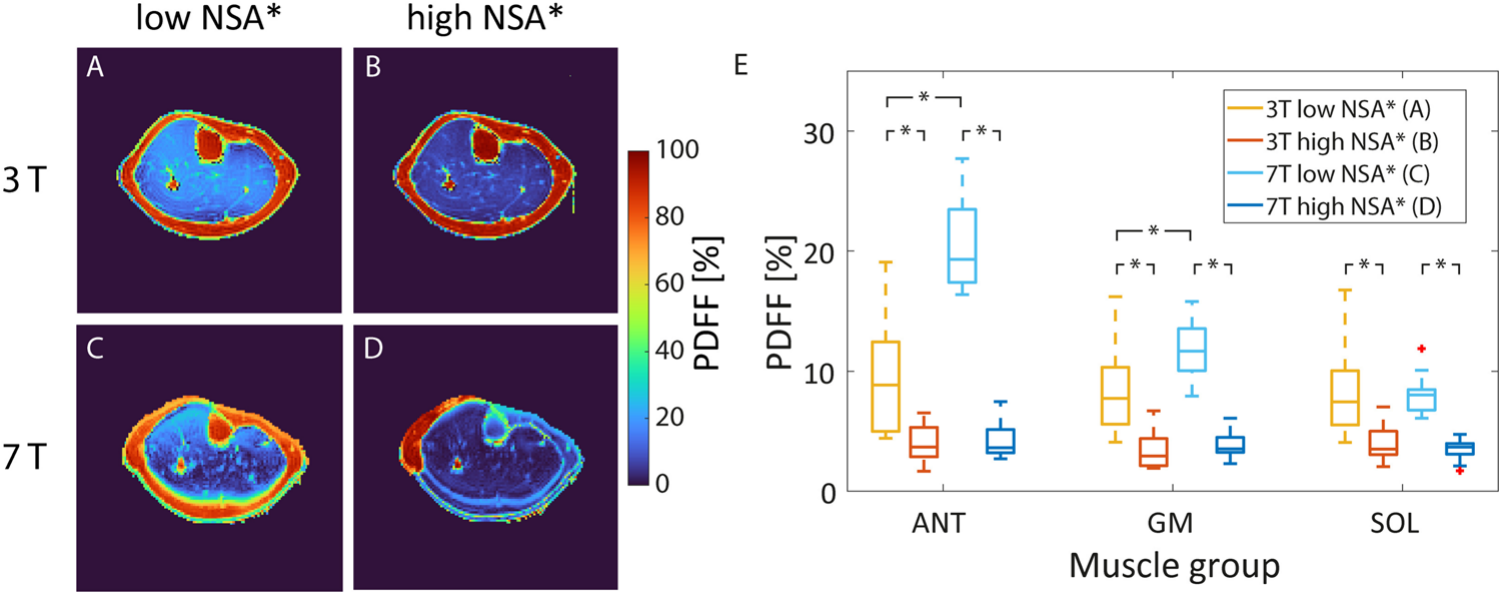
In vivo verification of the NSA* simulation at 3 T (**A**, **B**) and 7 T (**C**, **D**) acquired using phase encoding direction R-L. PDFF maps of a healthy female subject (top) depict an elevated PDFF at low NSA* echo times (**A**, **C**) compared to high NSA* echo times (**B**, **D**). At 7 T, fat–water swaps within the bone marrow and the subcutaneous fat appeared using high NSA* echo times with shorter TE_1_ without phase demodulation. Overall, the PDFF was significantly higher for low NSA* echo times than for high NSA* echo times at 3 T and 7 T (*n* = 12, E, marked with asterisk). In addition, the mean PDFFs for low NSA* echo times significantly differed between 3 T and 7 T in the ANT and GM muscles. In contrast, the mean PDFFs did not differ between 3 T and 7 T using high NSA* echo times

Corresponding in vivo results are shown in Fig. 4. In general, the measured PDFF within muscle tissue was significantly higher using low NSA* TEs than high NSA* TEs at both field strengths as well as for all evaluated muscle regions (*p* < 0.01). At 7 T, PDFF maps additionally showed a strong gradient from anterior to posterior within the muscle when using non-optimal TEs. Furthermore, chemical shift artifacts led to a strong blurring at tissue edges. The PDFF was significantly higher at 7 T than 3 T in the GM (*p* < 0.05) and ANT (*p* < 0.001) regions when using TEs corresponding to low theoretical NSA*. For high NSA* TEs, PDFF values in muscle tissue did not differ significantly between 3 T and 7 T. However, even when using high NSA* TEs, fat–water swaps within the subcutaneous fat tissue or bone marrow occurred in the PDFF maps acquired at 7 T. In contrast, none of the PDFF maps acquired using the high NSA* protocol at 3 T showed fat–water swaps.

**Fig. 5:**
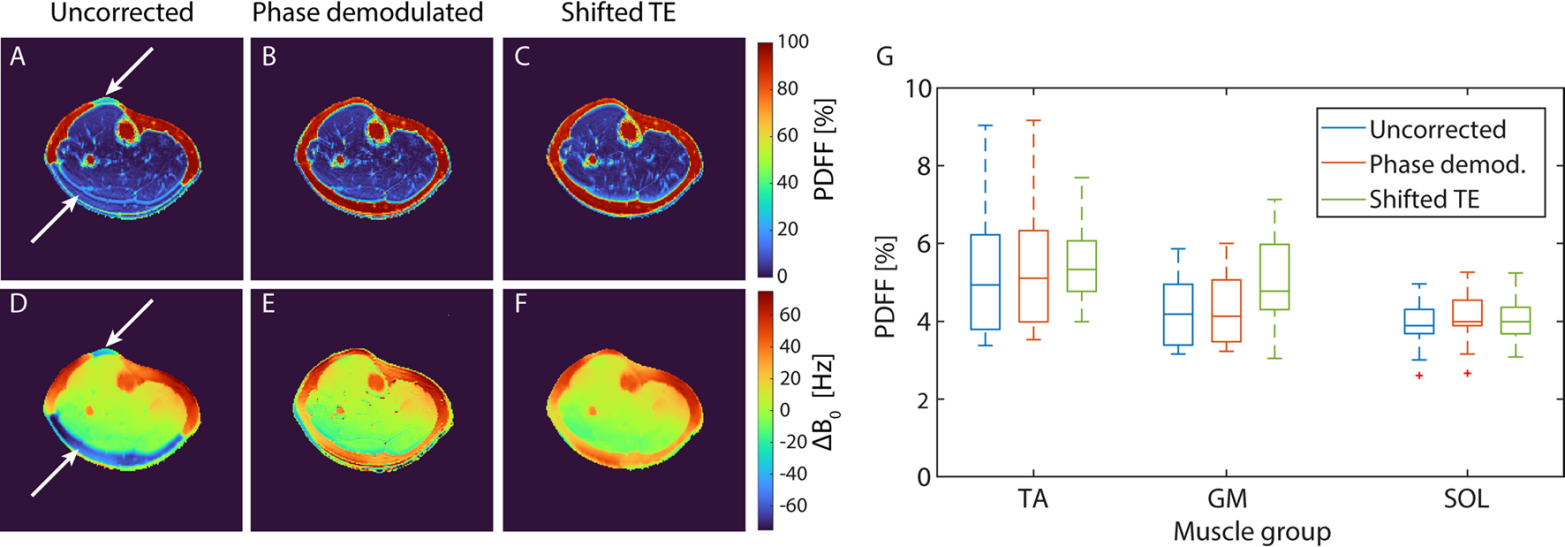
Impact of phase correction on PDFF maps acquired at 7 T. Exemplary PDFF maps (**A**–**C**) and corresponding field maps calculated by the Graph Cut algorithm (**D**–**F**) of the calf of a healthy female subject are shown. Without applying a phase correction, the field map depicts discontinuities within the subcutaneous fat (**D**, see white arrows), causing fat–water swaps in the corresponding PDFF map (**A**). Demodulation of the raw phase images using a precalculated B_0_ map acquired with optimized echo times (**B**) and shifting the echo times by 0.3 ms eliminated the swaps (**C**). Mean PDFF values within the individual muscle regions did not change significantly after correction of phase errors (G, *n* = 12)

To eliminate fat–water swaps within the subcutaneous fat tissue and bone marrow at 7 T, two different strategies were investigated: Demodulation of the raw phase using an additionally acquired B_0_ map as well as the use of TEs shifted by 0.3 ms (see Fig. 5). The shifted TEs corresponded to a slightly lower theoretical NSA* (see Fig. 2) but were still within a high-NSA* range. Without phase correction, the B_0_ maps created by the Graph Cut algorithm showed discontinuities leading to fat–water swaps in the PDFF maps of all 12 healthy subjects. Using shifted TEs, fat–water swaps still occurred in the PDFF maps of five out of 12 subjects. Phase demodulation removed fat–water swaps in all but four subjects. Overall, the severity of fat–water swaps was reduced in all 12 subjects using the proposed correction methods (exemplarily shown in Supplementary Information Fig. S6). Because shifting the TEs did neither require any additional measurement nor post-processing steps, this approach was chosen for further in vivo measurements. Exemplary $\text{T}_{2}^{*}$ maps produced by the Graph Cut algorithm for this optimized measurement scheme are shown in Supplementary Information Figure S7.

**Fig. 6:**
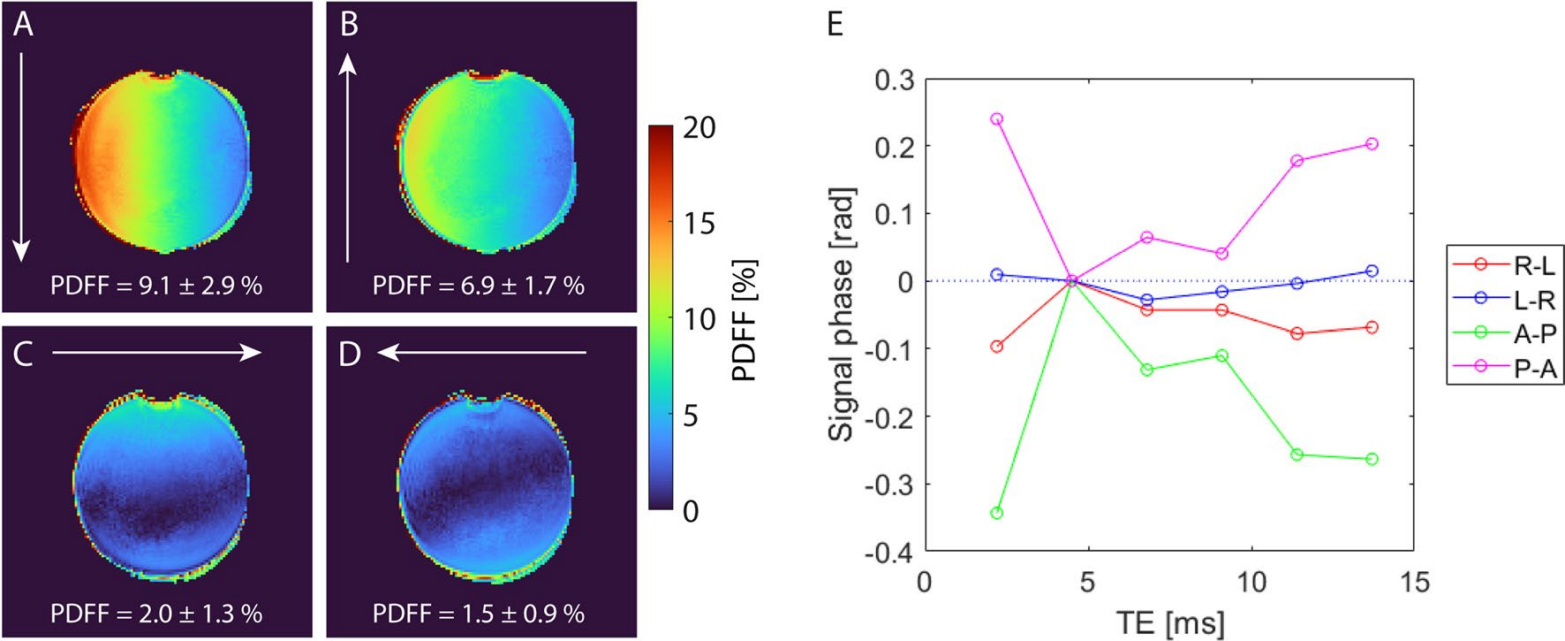
Influence of the phase encoding direction (white arrows) on the measured signal phase and determined PDFF maps for a water-only phantom at 7 T. Measurements were performed using four different phase encoding directions: Anterior–posterior (**A**), posterior-anterior (**B**), right-left (**C**), and left–right (**D**). The signal phase was demodulated for B_0_ inhomogeneities and normalized to zero at the second TE to increase comparability. Particularly for directions A-P and P-A, the signal phase strongly varied between the individual echo times, resulting in highly overestimated PDFF within the water-only phantom

**Fig. 7:**
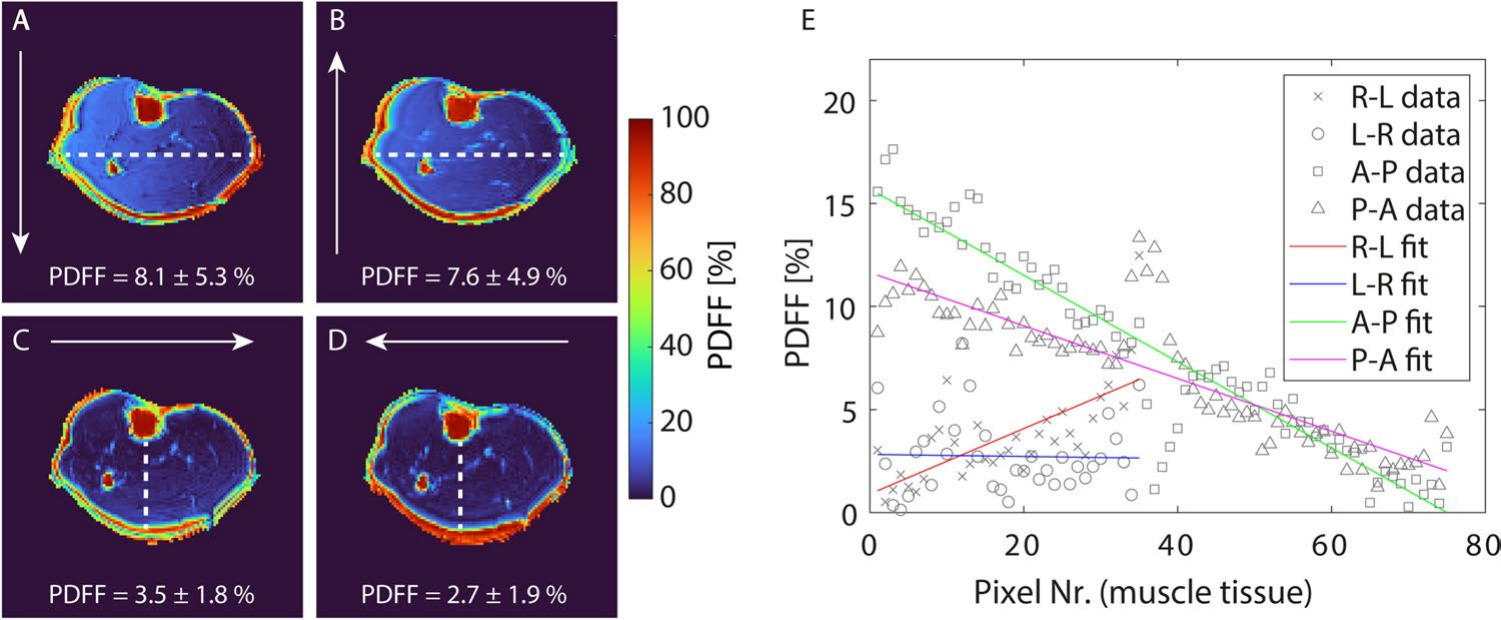
Influence of the phase encoding direction (white arrows) on the in vivo PDFF maps of a male healthy subject acquired at 7 T. A strong gradient in the PDFF from right to left was visible when using phase encoding directions A-P (**A**) and P-A (**B**). This gradient became particularly well visible when plotting the PDFF along a horizontal line (see white dashed lines) in the center of the calf (**E**). When using phase encoding direction R-L (**C**), a weaker gradient within the muscle ran from anterior to posterior (see vertical white dashed line). For opposite phase encoding L-R (**D**), the muscle tissue appeared more homogeneous and measured PDFF values did not vary along a vertical line. The PDFF values given within the figure represent the mean and standard deviation along the plotted lines

Even with successful reduction of fat–water swaps, a slight gradient of the PDFF across the muscle tissue was noted for the 7 T measurements. To investigate the origin of this artifact, a water-only phantom was examined. The impact of the phase encoding direction at 7 T on the signal phase after demodulation as well as the measured PDFF within this water-only phantom is depicted in Fig. 6. Phase encoding directions from anterior to posterior (A-P) and vice versa led to strong variations in the signal phase, resulting in an overestimation of the PDFF measured within the water-only phantom (9.1 ± 2.9% for A-P, 6.9 ± 1.7% for P-A, respectively). Phase encoding directions from left to right (L-R) and vice versa resulted in less deviant phase, and PDFF values better matched the expected value of zero (2.0 ± 1.3% for R-L, 1.5 ± 0.9% for L-R, respectively). Overall, the L-R direction showed best performance regarding phase effects and PDFF values. As for the water-only phantom, phase encoding directions A-P and P-A resulted in strong PDFF gradients within muscle tissue at 7 T, while phase encoding direction L-R minimized the bias and resulted in a homogeneous PDFF distribution over the entire healthy muscle tissue (see Fig. 7).

**Fig. 8:**
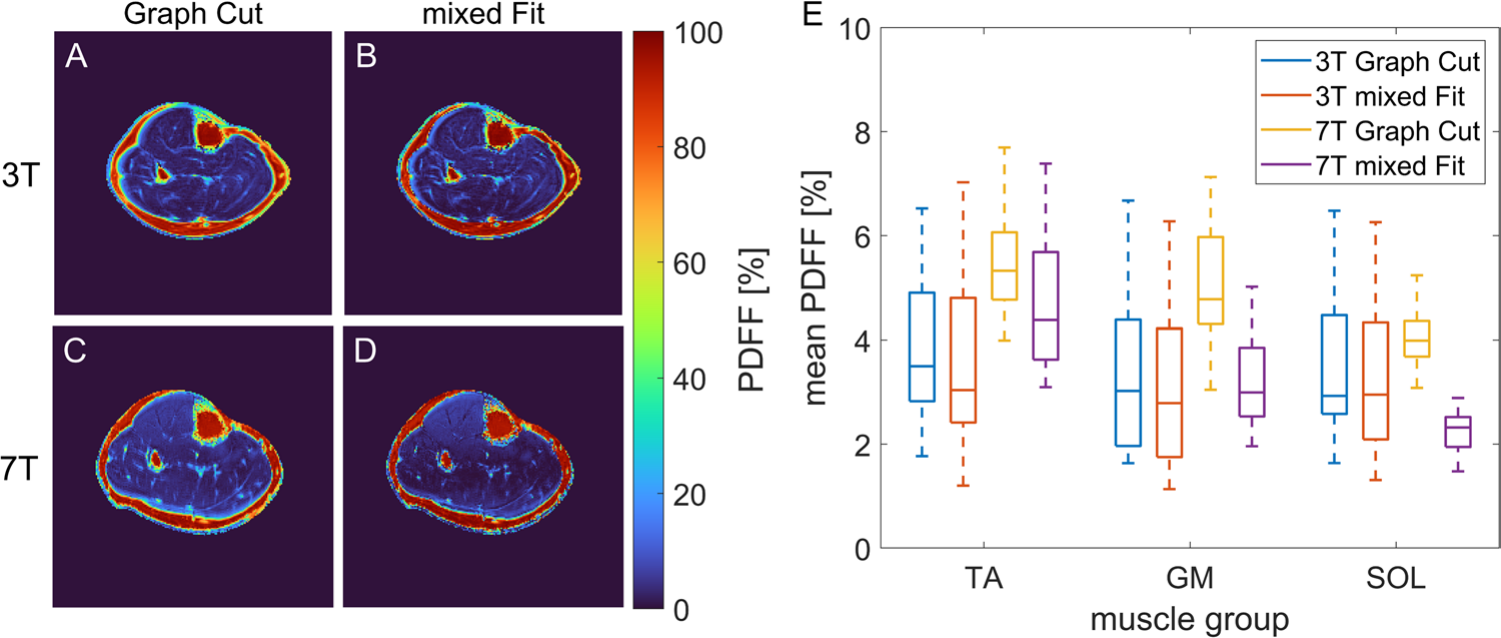
Effect of applying an additional voxel-wise mixed fitting approach after FWS by using the Graph Cut algorithm. In contrast to the Graph Cut algorithm, which is based on the full complex signal data, the mixed fitting regards only the magnitude information from the first echo while using the full complex data for all other echoes. At 3 T, PDFF maps using the mixed fitting (**B**) were comparable to using only the Graph Cut algorithm for PDFF estimation (**A**) as exemplarily shown for a male volunteer. This was confirmed by the mean PDFF values within the three muscle groups (TA, GM, SOL) which showed no relevant change over all twelve volunteers (**E**). At 7 T, using the mixed fitting reduced the mean PDFF values consistently across the muscle tissue. However, the variation of the PDFF values along the frequency encoding direction (anterior to posterior) (**C**) was still notable after applying the mixed fitting (**D**) leading to slightly increased PDFF values within the TA compared to the other muscle groups in some subjects for both methods

Additionally, we analyzed if using a mixed fit model as published by Hernando et al. [34] reduces the influence of the phase errors on the PDFF maps. At 3 T, the mixed fit model resulted in similar results to the Graph Cut algorithm (see Fig. 8). In contrast, at 7 T, the estimated PDFF across the entire muscle tissue was reduced when using the mixed fit model. However, the gradient of the PDFF along the frequency encoding direction was still apparent. Using the Graph Cut algorithm only lead to slightly higher mean PDFF values compared to 3T. Applying the mixed fit model lowered the estimated PDFFs, but the result was still inhomogeneous across the muscle tissue including values close to 0% in the central muscle region. As the mixed fit model did not improve the artifact at 7 T and partially led to unrealistic results, the Graph Cut approach was used for further calculation of the PDFF maps.

**Fig. 9:**
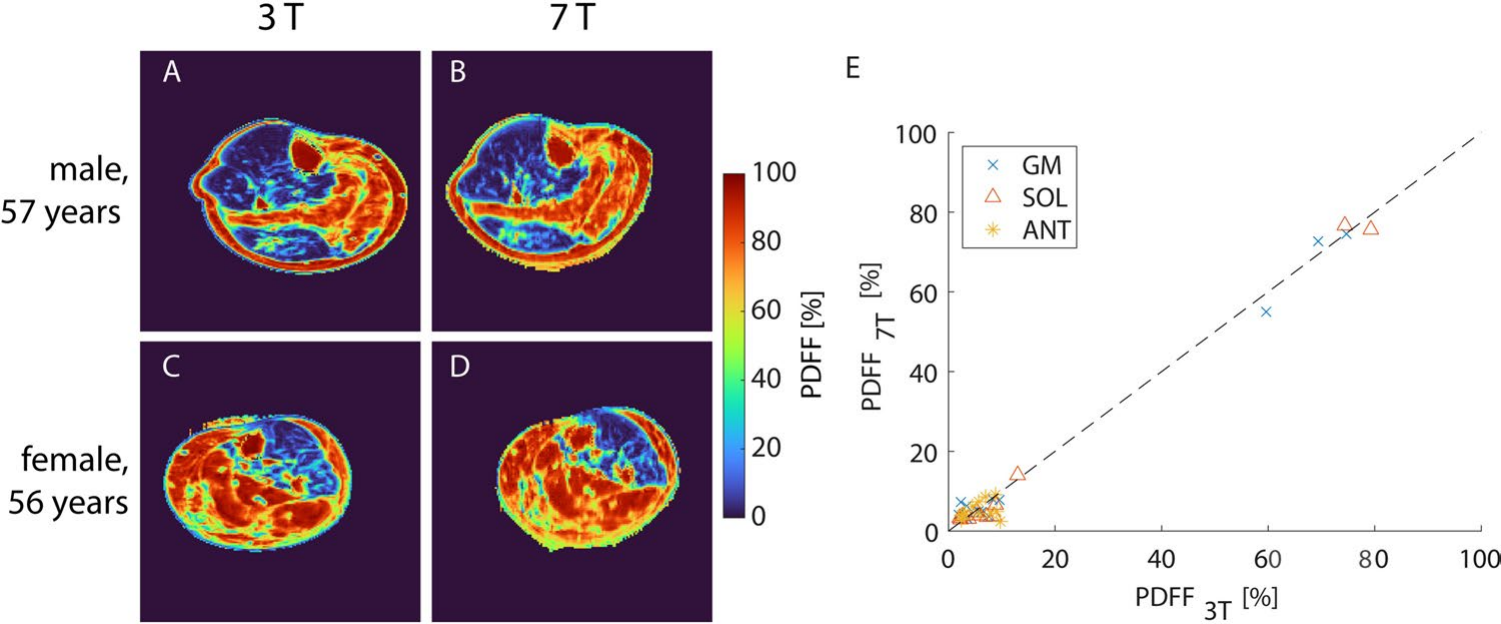
PDFF maps of two patients exhibiting hypokalemic periodic paralysis with severe fatty replacement of muscle tissue in the calf acquired using optimized parameters at 3 T (**A**, **C**) and 7 T (**B**, **D**) showed good qualitative agreement. However, blurring on tissue edges due to chemical shift was slightly higher at 7 T compared to 3 T. Quantitative PDFF results from all 12 subjects (9 healthy, 3 patients) for the three evaluated muscle groups (GM, SOL, ANT) showed a significant correlation between 3 and 7 T (**E**)

Finally, PDFF values measured at 3 T and 7 T in the same subjects were compared using the optimized acquisition parameters for 7 T (TE_1_ = 2.2 ms, ΔTE = 2.3 ms, phase encoding direction L-R). The qualitative impression of the PDFF maps acquired at 3 T and 7 T was in good accordance for both healthy subjects and patients exhibiting regions with increased fatty replacement of muscle tissue (see Fig. 9). The mean deviation of the PDFF values within the individual muscle ROIs between 3 and 7 T was 2.04%, reaching a maximum deviation of 7.32% in the anterior muscle segment of one subject. Furthermore, the results showed a significant correlation between PDFF values measured at the two field strengths (Spearman *R* = 0.62, *p* < 0.001). Regarding only the patient data, which included both low and high PDFF values, lead to a higher correlation (*R* = 0.95, *p* < 0.001), while for the volunteer only data with PDFF values < 10% a significant correlation was found only in the soleus muscle (*R* = 0.70, *p* = 0.043).

## Discussion

In this work, chemical-shift based FWS and PDFF determination of calf muscle tissue at 7 T was optimized using a vendor-provided GRE sequence with six equidistant echoes for image acquisition as well as a publicly available Graph Cut algorithm for post-processing of the complex MR signal. Sources of artifacts in PDFF maps acquired at 7 T were investigated, and potential strategies to remove these artifacts were evaluated. Quantitative PDFF results were compared to corresponding values measured at 3 T.

TEs for optimal sampling efficiency at 7 T were selected based on simulation of the theoretical NSA*. These simulations emphasized the need for optimized TEs for FWS at UHF as TE combinations leading to high theoretical NSA* at 7 T were very restricted. Phantom experiments as well as measurements of healthy human calf muscle tissue confirmed the NSA* simulation results. At 7 T, the mean deviations between the measured PDFF and the expected VFF of the peanut-oil/agarose phantoms using low NSA* TEs were twice as high as at 3 T. This was also confirmed by the in vivo results, where a significant overestimation of muscle PDFF followed from low NSA* TEs at 3 T, and even more severe at 7 T. The overestimation of low PDFF values and underestimation of high PDFF values observed for low NSA* TEs is related to the magnitude formulation of the PDFF calculation used in this work. This formulation projects all PDFF values to a range of 0% to 100%. Using a noise bias free formulation might instead present PDFF values < 0% or > 100%. Overall, these findings stress that choosing the TEs without regarding the NSA*, for instance by smallest possible ΔTE as has been suggested for multi-point Dixon acquisitions at lower field strength [26], cannot be recommended for UHF. Instead, the lowest achievable ΔTE that provides high NSA* should be chosen.

However, not only the TEs showed a strong impact on the quality of FWS at UHF. Also, the phase encoding direction considerably influenced the phase of the measured MR signal and thus, resulting PDFF maps. Phase deviations are most likely caused by eddy currents, which have been reported to cause biases in the FWS [34, 36, 37]. While gradient-induced eddy currents only depend on the gradient system but not on the field strength, vibrational eddy currents increase with the main magnetic field strength and therefore cause stronger artifacts at UHF [38]. In the PDFF maps, we observed a gradient perpendicular to the phase-encoding direction (i.e., in readout direction) at 7 T, whose strength varied depending on the chosen phase encoding direction. Using phase encoding directions L-R or R-L minimized the deviation of the phase signal and connected PDFF gradients in the calf. Similar misestimation of the PDFF due to phase errors has been observed at lower field strength before and could be solved by applying a mixed fitting approach that disregards the phase information of the first echo [34]. However, applying this approach to our data at 7 T did not resolve the artifact. Possibly, phase errors were not only apparent in the first echo but also across further TEs preventing the method to succeed.

The allowed minimal ΔTE with the used vendor sequence was bigger than the time of one phase cycle in between fat and water. This increases the risk of errors within the fieldmap estimates calculated by the FWS in regions of large field inhomogeneities, which can result in fat–water swaps in the water and fat images. This problem becomes more relevant at higher field strength due to the larger absolute field inhomogeneities and the sparser sampling of the phase oscillation considering comparable sequence limitations on ΔTE. Therefore, a correction of the signal phase was necessary to avoid fat–water swaps at 7 T. Misinterpretation of water and fat is a common challenge in FWS in the presence of large field inhomogeneities [10, 32, 33]. In this work, we evaluated the application of an additionally acquired B_0_ map for phase demodulation before FWS as well as the use of slightly longer TEs to mitigate fat–water swaps at 7 T. Demodulation with an initial estimation of the B_0_ map reduces the effective range of off-resonances observed by the FWS algorithm and can thereby improve the stability of complex-based FWS algorithms that assume smoothness of the B_0_ map. This effect was already described for using a demodulation with a susceptibility-based initial guess of the field map [32, 33]. Both correction methods, the phase demodulation and the longer TEs, led to comparable results, removing fat–water swaps in a majority of data sets (phase demodulation: no swaps in 8/12; shifted TEs: no swaps in 7/12), and reducing their severity in the remaining ones. A combination of both led to even better results (no swaps in 10/12 data sets). The performance of the phase demodulation was limited by the quality of the calculated B_0_ maps. Due to low SNR, the region of the tibia was masked during the calculation of the B_0_ map, explaining remaining fat–water swaps in this area. If the PDFF signal within the tibia is relevant for an application, using a phase demodulation with a different B_0_ mapping or estimation approach might further reduce fat–water swaps in this region. At 3 T, phase correction was not performed as fat–water swaps occurred in none of the uncorrected data sets. An advantage of using longer TEs compared to phase demodulation is that this approach neither prolongs the acquisition time nor requires additional post-processing steps. However, resulting PDFF maps appeared slightly blurrier than PDFF maps acquired with shorter TEs. Thus, performing a phase demodulation on data acquired with shorter TEs might be advantageous if a slightly longer total measurement time is acceptable.

For post-processing of phantom and in vivo data, multi-peak fat spectra models of peanut oil and subcutaneous adipose tissue were determined. The observed relative peak frequencies were in good agreement with the literature [39, 40]. The shift of the main fat resonance relative to water was noticeably smaller in vivo (– 3.24 ppm) than in phantom (– 3.51 ppm). The observed fat–water shift in subcutaneous fat tissue is in good agreement with data shown in literature [40]. The deviations to the phantom are most likely explained by variations in pH and temperature, which influence the resonance frequency of water while having negligible effect on fat signal [41]. For instance, an increase in temperature of 1 °C was reported to result in a decrease of the fat–water shift of 0.01 ppm [42]. Nevertheless, we were able to successfully perform FWS with the used mean fat peak values acquired in the subcutaneous fat of healthy subjects in all healthy volunteers as well as in patients with hypokalemic periodic paralysis, a muscular channelopathy.

In this study, a magnitude-based definition of the PDFF was used. While this definition is known to introduce some noise bias for very low or high PDFF [12], it proved to be more robust against the phase errors observed within our 7 T data than complex-valued definitions.

The mean PDFF values in the muscle ROIs at 3 T and 7 T mostly showed a good agreement with a mean deviation of 2%. However, for one single case, deviations reached up to 7.3%. Higher deviations were mainly observed in the regions that had shown phase issues at 7 T like the anterior muscle region and the gastrocnemius muscle. Hence, the precision of the PDFF estimation might be further improved by applying an additional correction for phase errors before the FWS. Further deviations might be caused by inconsistencies in drawing of the ROIs and by noise bias for low PDFFs. At 3T, patient data was acquired with a different coil and scanner setup than the healthy volunteer data due to organizational issues. However, phantom experiments revealed no bias, for instance due to differences in SNR, in the PDFF estimation with both coil setups (Supplementary Information Figure S8). As the SNR increases with field strength, we expect the potential influence of the noise bias to be even lower at 7 T though the used knee coil showed an inhomogeneous receive profile with lower signal in the anterior part of the leg. To mitigate a potential T_1_ bias, a small flip angle of 3° was chosen (compare Supplementary Information Fig. S1). At 7 T, a spatial variation of the flip angle up to about 4° in the center region of the calf could occur due to B_1_ inhomogeneity. However, according to the signal equation the expected T_1_ bias is still minor. Overall, we observed a significant correlation of the PDFF values observed at 3 T and 7 T, and pathologic fatty replacement of the muscle tissue could be depicted comparably at both field strength. However, to better assess the correlation and accuracy of the PDFF estimation, evaluation of a larger patient cohort providing PDFF values over the full range from 0 to 100% would be necessary.

Overall, the choice of TEs maximizing the NSA* under practical conditions was restricted by hardware and sequence limitations. In theory, using even shorter TE_1_ and ΔTE could lead to a better noise efficiency and reduce the risk of fat–water swaps and thus improve FWS. However, using shorter TEs might increase the influence of eddy current-related artifacts. The minimum achievable ΔTE could be reduced using bipolar readout gradients, two acquisitions with three echoes each, or using a sequence scheme enabling interleaved acquisition of two echo trains with three echoes each. By switching from monopolar to bipolar readout the shortest achievable echo spacing with the used vendor sequence was still way longer than the optimal echo spacing, especially at 7 T. In addition, using a bipolar readout or splitting the echoes over multiple (interleaved) acquisitions introduces additional phase errors that must be corrected [37, 43, 44]. Typical correction approaches require the measurement of additional k-space lines, for instance with the opposite gradient polarity, to calculate the phase errors. Thus, all three options cannot be performed with a basic GRE sequence, but require the implementation of more advanced MRI sequences or the use of additional reference scans, which is impractical in a clinical study setting. Overall, our proposed protocol used a commonly available, vendor-provided spoiled GRE sequence and required a total acquisition time of approx. 1 min, which allows easy integration in measurement protocols of clinical studies.

## Conclusion

Even with restricted echo time choices of vendor-provided sequences, FWS and PDFF determination in calf muscle tissue is feasible at 7 T using a chemical-shift based approach with optimized acquisition and post-processing parameters, resulting in comparable muscular PDFF values as measured at 3 T.

## Supplementary Information

Below is the link to the electronic supplementary material.

Supplementary file1 (PDF 1314 KB)

## Data Availability

The data that support the findings of this study are available from the corresponding author, Tkotz K, upon reasonable request.
