# Supplementary material for: Parameter optimization for proton density fat fraction quantification in skeletal muscle tissue at 7 T
Source: MAGMA. 2024 Aug 6;37(6):969–81. doi: 10.1007/s10334-024-01195-2 (PMC11582128; doi:10.1007/s10334-024-01195-2)
Supplement: Supplementary file 1 — Supplementary file1 (PDF 1314 KB) [file 10334_2024_1195_MOESM1_ESM.pdf]

Supplementary Information

To the manuscript

Parameter optimization for proton density fat fraction quantification in skeletal muscle tissue at 7 T

By Katharina Tkotz[1], Paula Zeiger, Jannis Hanspach, Claudius S. Mathy, Frederik B. Laun, Michael Uder, Armin M. Nagel, Lena V. Gast

Submitted to Magnetic Resonance Materials in Physics, Biology and Medicine

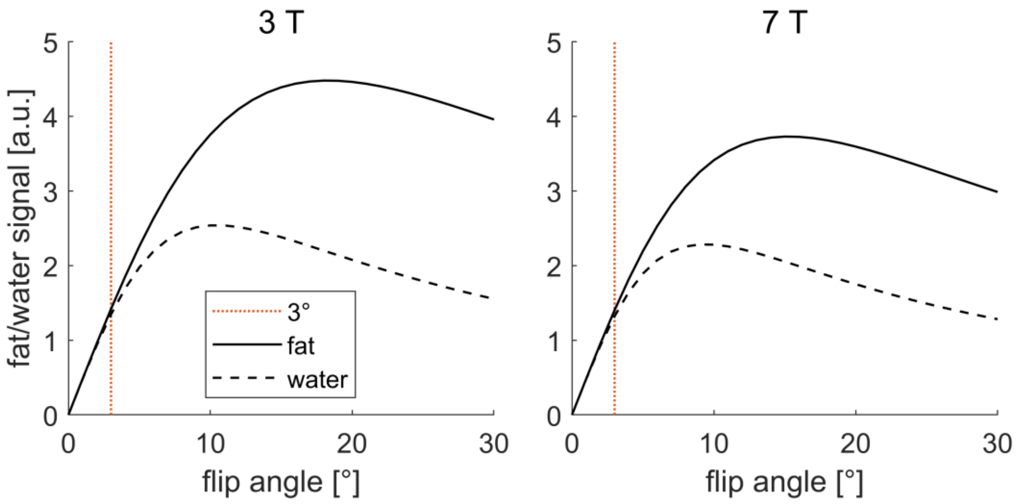

Supplementary Information Fig. S1 Simulation of the signal equation neglecting T_2* for fat and water signal for 3T and 7T for different flip angles α

$$S_{\mathrm{f/w}} = \frac{M_{\mathrm{f/w}} \left(1 - \exp\left(-\frac{TR}{T_{1\mathrm{f/w}}}\right)\right) \sin\alpha}{\left(1 - \exp\left(-\frac{TR}{T_{1\mathrm{f/w}}}\right)\right) \cos\alpha}.$$

The magnetization of fat and water was assumed to be equal $M_f = M_w$with arbitrary magnitude. The simulation was performed for a TR of 21 ms as used for the measurements, and with T_1 values for subcutaneous fat ($T_{1f}^{3T} = 404$ ms, $T_{1f}^{7T} = 583$ ms) and within the gastrocnemius muscle ($T_{1w}^{3T} = 1256$ ms, $T_{1w}^{7T} = 1553$ ms) taken from literature [1]. The used flip angle of 3° is marked by the dotted red line. As fat and water signal magnitudes correspond well, the T_1 bias is expected to be negligible for this flip angle at both field strengths

1. Jordan CD, Saranathan M, Bangerter NK, Hargreaves BA, Gold GE (2013) Musculoskeletal MRI at 3.0 T and 7.0 T: a comparison of relaxation times and image contrast. Eur J Radiol 82 (5):734-739.

[1] katharina.tkotz@uk-erlangen.de, Institute of Radiology, University Hospital Erlangen, Friedrich-Alexander-Universität Erlangen-Nürnberg (FAU), Erlangen, Germany

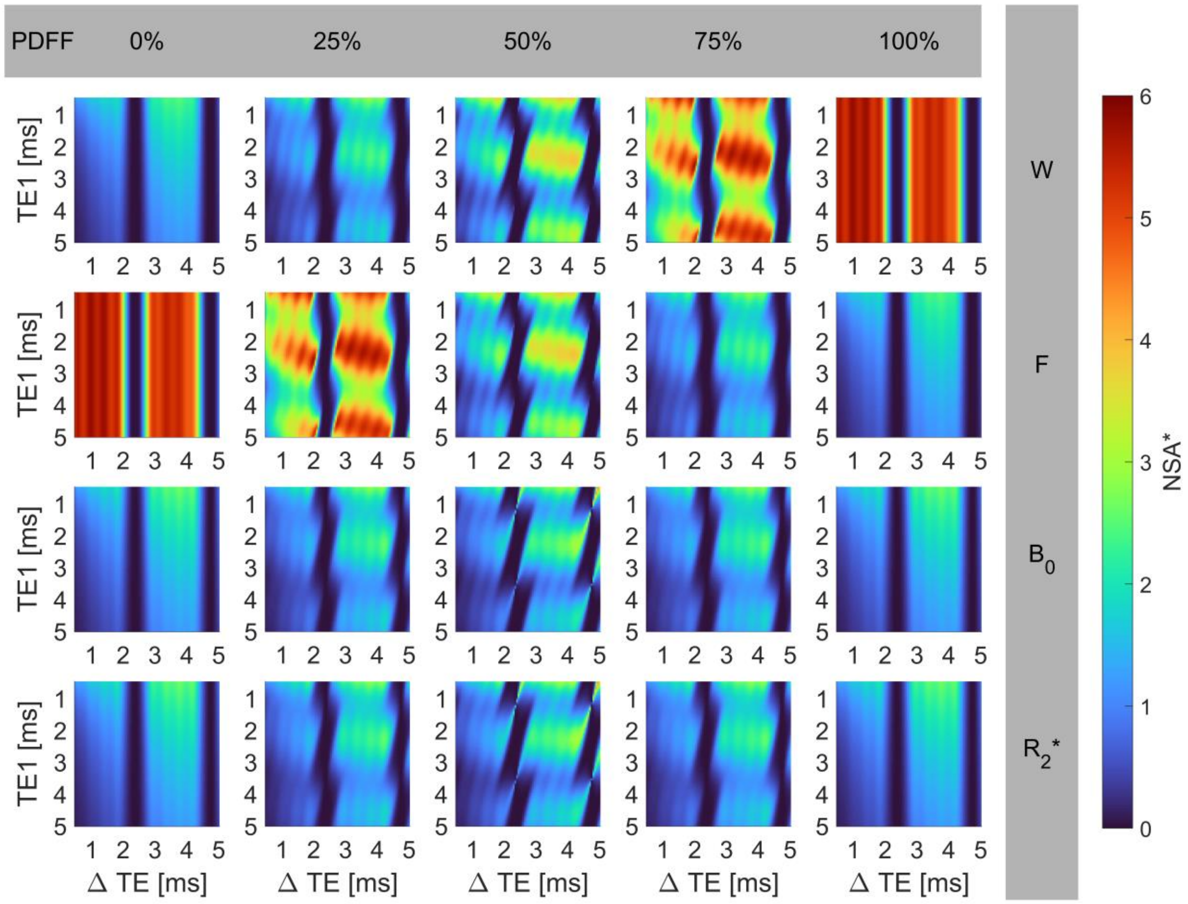

Supplementary Information Fig. S2 Simulated NSA* at B_0 = 3 T assuming a single-peak fat model with dominant fat peak at –3.3 ppm and R_2^* = 50 Hz shown in dependence of initial echo time (TE1) and echo time difference (ΔTE) from 0.5 ms to 5 ms. Results are shown for PDFFs ranging from 0% to 100% and for all signal model parameters: W: NSA* results for estimating the magnitude of the water-only signal which is equal to the results for the phase of the water signal; F: NSA* results for estimating the magnitude of the fat-only signal which is equal to the results for the phase of the fat signal; B_0: NSA* results for estimation of the off-resonance; R_2^*: NSA* results for estimation of R_2^*

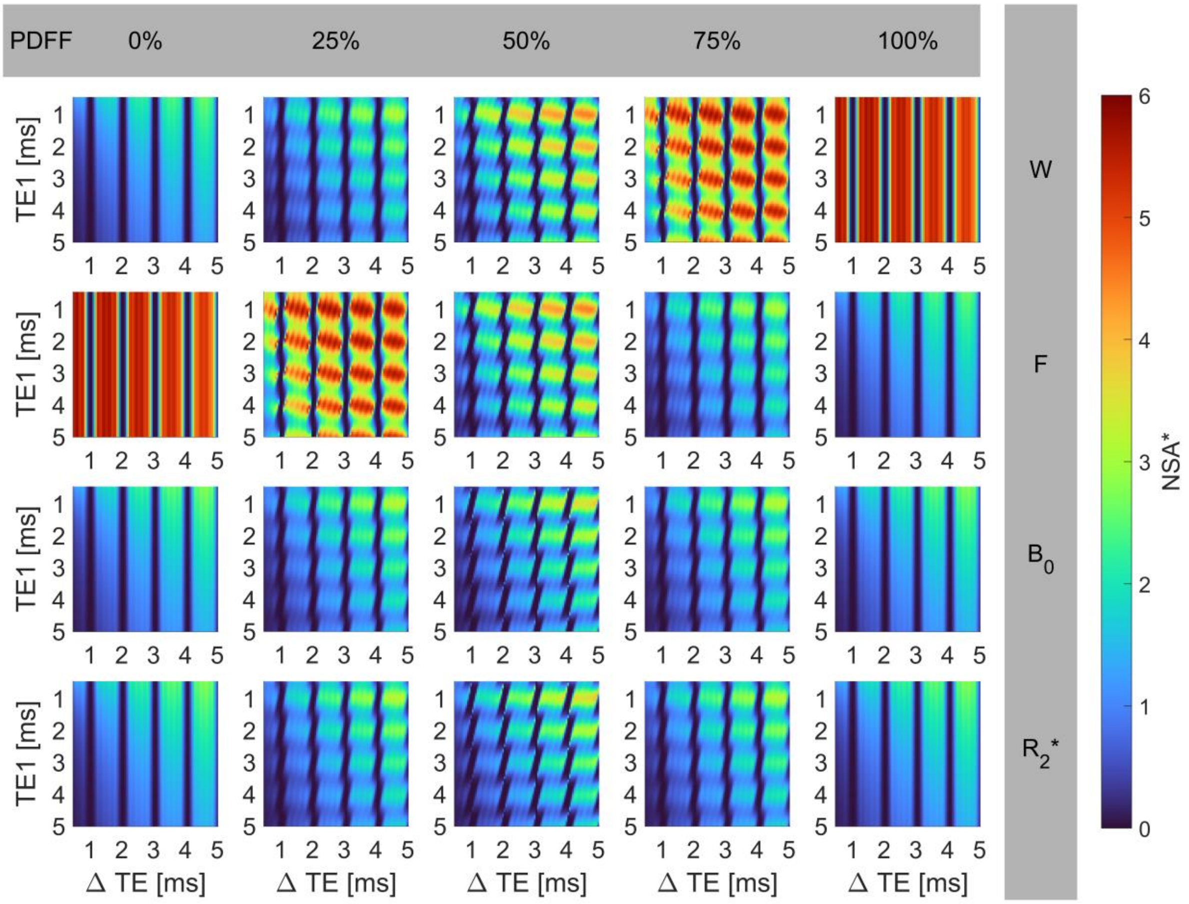

Supplementary Information Fig. S3 Simulated NSA* at B_0 = 7 T assuming a single-peak fat model with dominant fat peak at –3.3 ppm and R_2^* = 50 Hz shown in dependence of initial echo time (TE1) and echo time difference (ΔTE) from 0.5 ms to 5 ms. Results are shown for PDFFs ranging from 0% to 100% and for all signal model parameters: W: NSA* results for estimating the magnitude of the water-only signal which is equal to the results for the phase of the water signal; F: NSA* results for estimating the magnitude of the fat-only signal which is equal to the results for the phase of the fat signal; B_0: NSA* results for estimation of the off-resonance; R_2^*: NSA* results for estimation of R_2^*

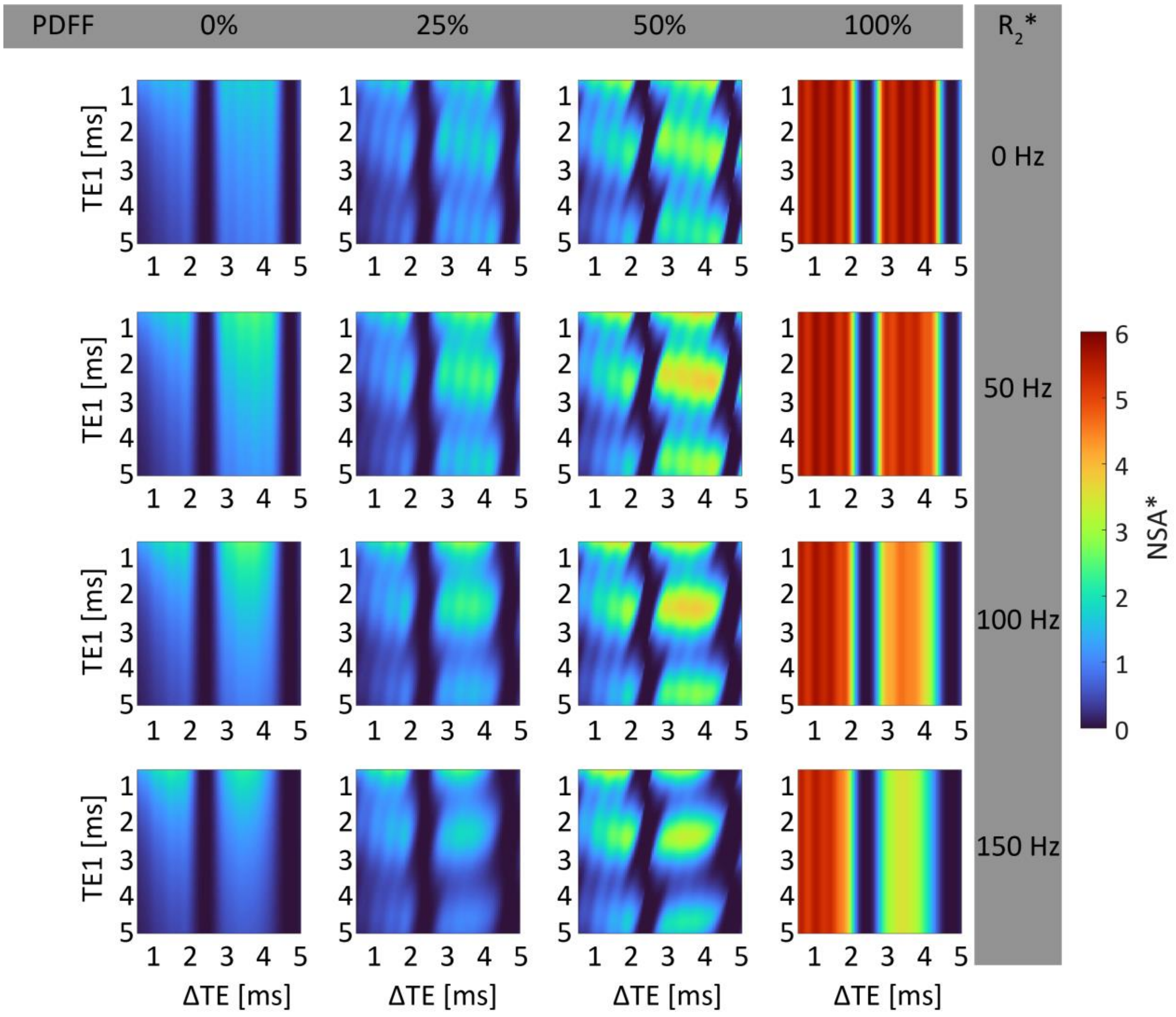

Supplementary Information Fig. S4 Simulated NSA* for estimates of water-only images by FWS in dependence of initial echo time (TE_1) and echo time difference (ΔTE) from 0.5 ms to 5 ms at $B_0 = 3$ T. Simulations were performed assuming a single-peak fat model with the dominant fat peak at –3.3 ppm. The relaxation rate R_2^* was varied from 0 Hz to 150 Hz and the PDFF from 0% to 100%. For increasing R_2^* values, the areas with high NSA* values are reshaped and the maximum achievable NSA decreases

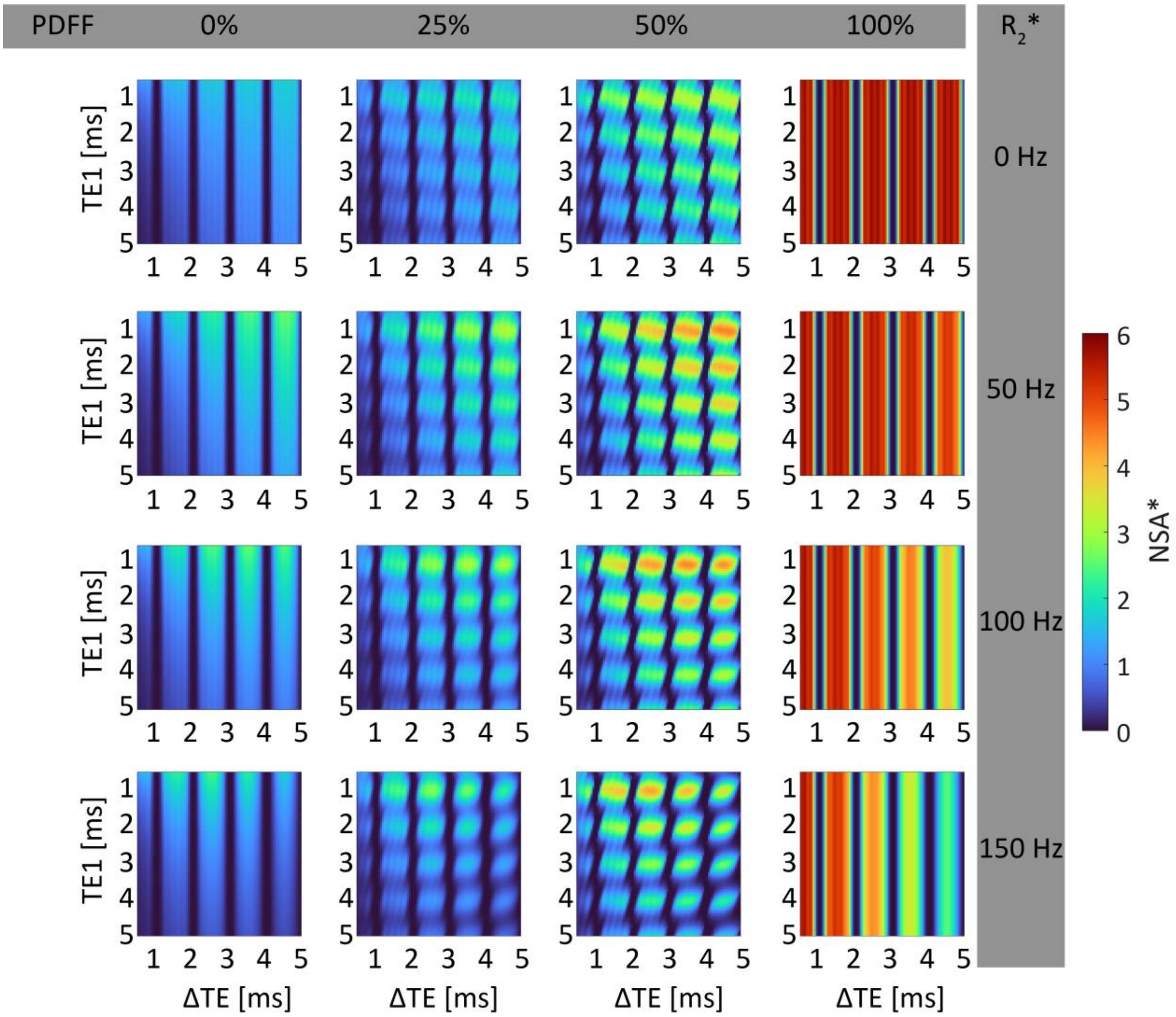

Supplementary Information Fig. S5 Simulated NSA* for estimates of water-only images by FWS in dependence of initial echo time (TE_1) and echo time difference (ΔTE) from 0.5 ms to 5 ms at $B_0 = 7$ T. Simulations were performed assuming a single-peak fat model with the dominant fat peak at –3.3 ppm. The relaxation rate R_2^* was varied from 0 Hz to 150 Hz and the PDFF from 0% to 100%. For increasing R_2^* values, the areas with high NSA* values are reshaped and the maximum achievable NSA decreases

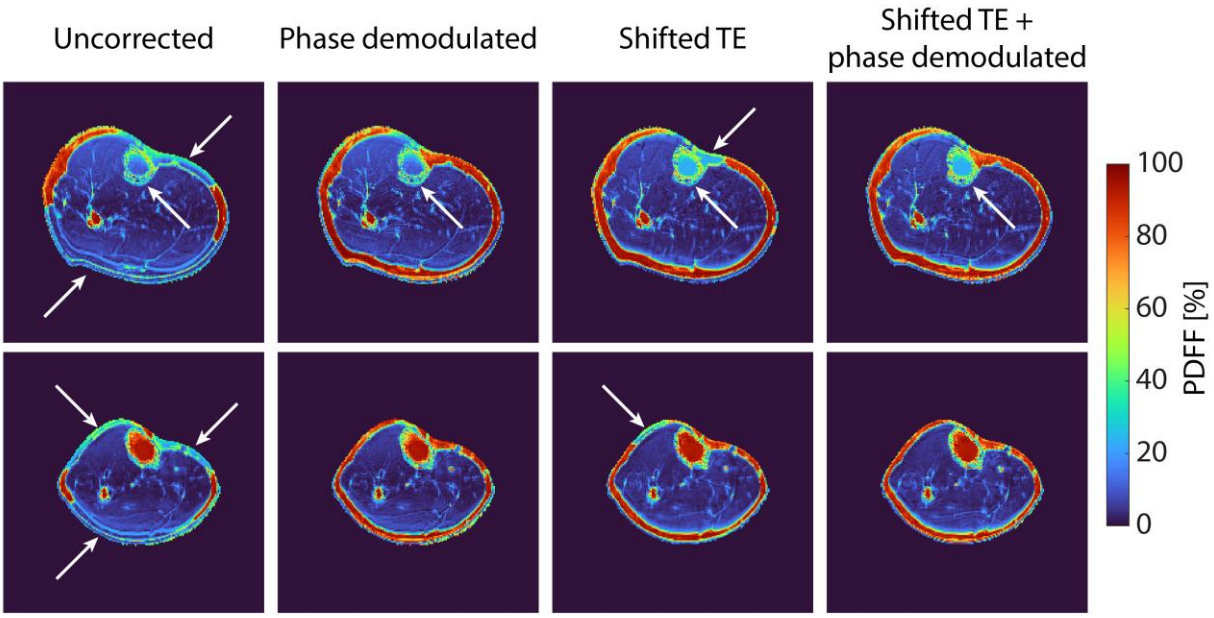

Supplementary Information Fig. S6 PDFF maps of two exemplary subjects, in which fat-water swaps were present before and after phase correction. While fat-water swaps occurred in the subcutaneous fat tissue and/or bone marrow of uncorrected PDFF maps of all 12 examined healthy subjects, the applied phase correction methods reduced the number and severity of fat-water swaps. For 5 volunteers, minor fat-water swaps remained after shifting the TEs by 0.3 ms, while demodulating the raw phase with an additionally acquired B_0 map removed most of the fat-water swaps. Performing a phase demodulation on the raw phase of the data acquired with shifted TEs resulted in further minor improvements

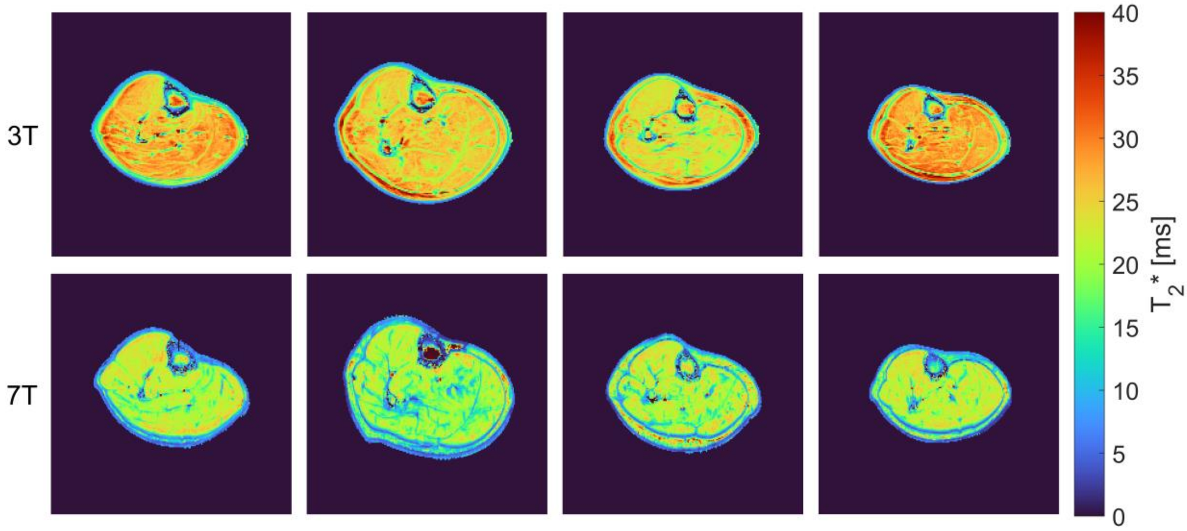

Supplementary Information Fig. S7 T_2^* maps produced by the Graph Cut FWS algorithm for the measurements at 3 T and at 7 T with the longer TE_1 shown for four exemplary healthy volunteers (2 male, 2 female). As expected T_2^* values at 7 T were lower than at 3 T consistently across all volunteers

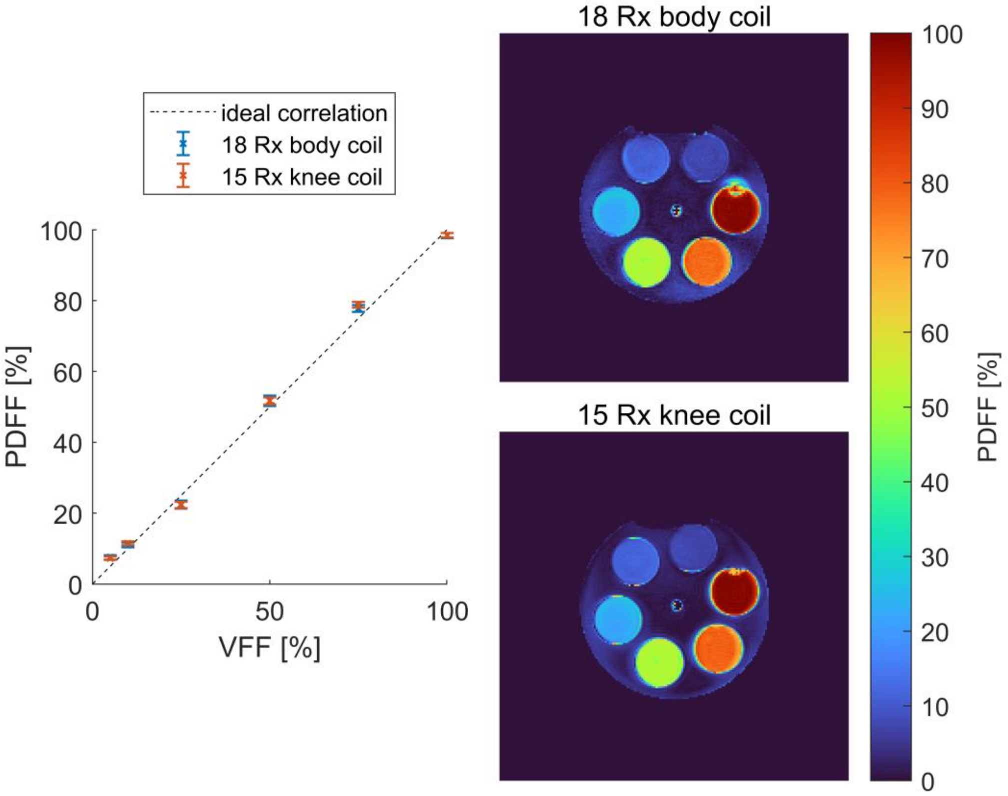

Supplementary Information Fig. S8 Comparison of the PDFF estimation in phantom with the two coil setups used at 3T: 18 Rx body coil + spine array, and 1Tx/15Rx knee coil. Though the image impression showed slightly higher SNR when using the knee coil, no bias in the mean PDFF values between both setups could be observed
